# Supplementary material for: Regulation of Structure and Quality of Dried Noodles by Liquid Pre-Fermentation
Source: Foods. 2021 Oct 11;10(10):2408. doi: 10.3390/foods10102408 (PMC8535864; doi:10.3390/foods10102408)
Supplement: Supplementary file 1 [file foods-10-02408-s001.zip › foods-1397845-supplementary.pdf]

Table S1. Detection results of volatile substances of dried noodles under different fermentation time and amount of yeast added

	Retention time (min)	Volatile compound	Relative content (%)									
			0 h-0.0%	1 h-0.2%	1 h-0.5%	1 h-1.0%	3 h-0.2%	3 h-0.5%	3 h-1.0%	6 h-0.2%	6 h-0.5%	6 h-1.0 %
Alcohols												
1	24.957	Tetradecanol	-	-	-	-	1.85	-	-	-	-	-
2	29.844	Lauryl alcohol	-	-	-	-	1.25	-	-	-	-	-
3	29.875	2- butyl -1- octanol	1.07	-	-	-	-	-	-	-	-	0.50
Aldehydes												
1	3.149	N-hexanal	1.81	-	-	-	-	-	-	-	-	-
2	8.586	Phenyl aldehyde	2.48	14.77	9.32	-	5.91	6.05	4.68	6.24	2.06	2.09
3	8.706	(Z)-2- heptene aldehyde	0.68	-	-	-	-	-	-	-	-	-
4	13.645	Nonanal	2.33	-	-	9.24	-	-	-	5.88	2.58	-
5	16.888	Decanal	6.10	-	-	-	-	-	-	-	0.72	-
6	32.345	Stearaldehyde	-	-	-	-	-	-	-	-	-	1.39
Esters												
1	9.868	Ethyl caproate	-	-	-	-	-	-	-	-	-	0.98
2	24.163	Tetradecyl methoxyacetate	-	-	-	-	2.56	2.03	1.93	-	4.70	-
3	24.183	Pentadecyl methoxyacetate	-	4.06	2.07	-	1.09	1.42	2.06	-	-	-
4	24.972	Cyclobutyl tetradecyl oxalate	-	-	-	3.41	-	-	-	-	0.30	-
5	25.034	Propyltetradecyl sulfite	-	-	-	2.68	1.28	-	-	-	0.71	-
6	25.169	Methoxy tridecane acetate	-	5.17	2.36	-	4.01	3.25	2.51	1.36	1.01	-
7	33.248	1,	-	-	3.13	-	2.06	5.99	-	2.66	14.85	11.3

		2-benzodicarboxylic acid bis (2-methylpropyl) ester										1
8	33.481	N-octyl phthalate	0.98	-	-	-	-	-	-	-	-	-
9	34.623	1, 2-phenyldicarboxylate butyloctyl ester	-	-	2.06	-	-	-	-	-	-	-
10	34.706	Ethyl hexadecenoate	-	-	-	3.34	-	-	-	-	2.47	8.32
11	34.707	Dibutyl phthalate	-	-	-	-	1.87	-	-	-	2.38	19.34
12	34.976	Ethyl hexadecanoate	-	-	1.89	8.87	1.10	-	-	-	12.98	10.18
13	36.984	Ethyl linoleate	-	-	-	7.87	-	-	-	-	-	-
14	36.994	Ethyl octadecadienoate	-	-	-	-	2.22	-	-	-	6.11	6.72
15	39.734	Butyl octadecanoate	-	-	-	-	-	-	-	-	2.24	-
Carbonic acids												
1	33.077	Pentadecane acid	-	-	-	-	-	-	-	-	-	0.42
2	34.530	Palmitic acid	-	-	-	-	-	-	-	2.73	3.83	-
Aromatics												
1	16.157	Naphthalene	0.83	7.29	-	-	-	3.70	4.51	3.44	-	2.37
Olefins												
1	10.963	Dexilionene	-	-	-	-	-	1.10	1.46	1.12	0.66	-
2	16.691	Tetramethylhexadecane	-	-	11.57	-	-	-	-	-	-	1.11
3	16.956	Undecane	-	-	-	-	-	1.39	1.78	-	-	-
4	16.961	Dodecane	16.78	13.80	-	-	-	13.40	9.59	4.21	-	1.07
5	19.571	Trimethylkwai	-	-	-	14.53	-	-	-	7.37	-	-
6	19.711	Tridecane	43.24	25.25	29.59	-	21.29	19.57	18.59	-	8.78	6.45
7	20.593	Dodecylcyclohexane	1.01	5.45	4.35	6.37	4.15	3.90	2.81	3.32	5.23	10.6

												0
8	21.693	1-iodo2-methylundecane	-	-	-	3.46	-	7.33	6.62	5.00	-	-
9	22.233	(E) - 9 - oleic	-	-	-	1.04	-	-	-	-	-	-
10	22.274	Tetradecene	-	-	-	-	-	-	-	-	-	0.30
11	22.502	9-methyl-decadecane	-	-	9.90	-	-	-	3.28	-	-	-
12	22.518	Tetradecane	12.58	-	17.96	11.68	9.32		8.46	6.11	5.10	2.62
13	22.782	Hexadecane	-	19.11	5.31	7.66	10.21	9.89	5.17	6.12	5.70	2.65
14	23.805	Nonyl-cyclopentane	-	-	-	-	-	-	3.19	-	-	-
15	23.905	(1-methylethyl)-cycloundecane	-	-	-	-	-	-	-	2.03	-	-
16	24.163	2,6,10-trimethyl-dodecane	-	-	-	-	-	-	-	3.28	-	-
17	24.168	3,8-dimethyl-undecane	-	-	-	-	-	-	1.71	-	-	-
18	24.946	1-tridecene	-	-	-	-	-	-	-	-	0.82	0.44
19	24.967	Pentadecane	-	-	-	-	-	-	-	6.18	4.04	-
20	24.977	Trichlorodocosyl-silane	-	-	-	-	-	-	2.41	-	-	-
21	25.128	10-methyldecadecane	-	-	-	5.11	-	-	-	-	-	-
22	25.953	1-chloro-heptacosane	-	-	-	-	-	-	-	0.77	1.63	-
23	26.088	Trimethyl-tetracontane	-	-	-	-	-	-	-	3.53	-	-
24	26.321	Tetratetracontane	-	-	-	-	6.25	-	4.16	1.94	3.32	-
25	26.788	(E)-2-tetradecene	-	-	-	3.87	-	4.78	-	-	-	1.91
26	26.809	(E)-2-tetradecene	-	2.99	-	-	-	-	-	-	-	-
27	26.928	3-methyl-pentadecane	-	-	-	-	7.67	-	-	-	-	-
28	27.312	Cetene	-	-	-	-	-	-	-	-	0.80	0.62
29	27.463	[(dodecyloxy)methyl]-oxirane	-	-	-	-	-	-	-	1.15	-	-

30	27.463	1-(ethenyloxy)-octadecane	-	-	-	1.72	-	-	-	-	-	-
31	28.905	1-chloro-octadecane	-	-	-	-	1.69	1.54	1.51	-	-	-
32	28.978	Undecyl-cyclopentane	-	-	-	2.56	-	1.78	-	-	-	-
33	28.993	Cyclohexadecane	-	2.10	-	-	-	-	-	2.04	-	-
34	30.016	Heptadecane	-	-	-	-	-	-	-	-	3.63	0.71
35	30.15	Tetramethylpentadecane	-	-	-	-	-	2.04	-	-	-	1.11
36	30.265	Octadecane	-	-	-	-	2.79	-	-	-	-	-
Furans												
1	7.72	2-pentylfuran	-	-	-	-	7.66	9.24	5.30	9.91	2.28	4.68
Others												
1	16.411	1-methylene -1H-indene	-	-	-	4.01	-	-	-	-	-	-

- not detected.
